# Moving from formative research to co-creation of interventions: insights from a community health system project in Mozambique, Nepal and Peru

**DOI:** 10.1136/bmjgh-2018-001183

**Published:** 2018-11-16

**Authors:** David Beran, Maria Lazo-Porras, Maria Kathia Cardenas, François Chappuis, Albertino Damasceno, Nilambar Jha, Tavares Madede, Sarah Lachat, Silvana Perez Leon, Nathaly Aya Pastrana, Maria Amalia Pesantes, Suman Bahadur Singh, Sanjib Sharma, Claire Somerville, L Suzanne Suggs, J Jaime Miranda

**Affiliations:** 1 Division of Tropical and Humanitarian Medicine, University of Geneva and Geneva University Hospitals, Geneva, Switzerland; 2 CRONICAS Centre of Excellence in Chronic Diseases, Universidad Peruana Cayetano Heredia, Lima, Peru; 3 Division of Tropical and Humanitarian Medicine, University of Geneva, Geneva, Switzerland; 4 Faculty of Medicine, Eduardo Mondlane University, Maputo, Mozambique; 5 B. P. Koirala Institute of Health Sciences, Dharan, Nepal; 6 BeCHANGE Research Group, Institute of Public Communication, Università della Svizzera italiana, Lugano, Switzerland; 7 Gender Centre, Graduate Institute of International and Development Studies, Geneva, Switzerland; 8 Swiss School of Public Health, Zürich, Switzerland; 9 School of Medicine, Universidad Peruana Cayetano Heredia, Lima, Peru

**Keywords:** study design, health services research

## Abstract

Different methodological approaches for implementation research in global health focusing on how interventions are developed, implemented and evaluated are needed. In this paper, we detail the approach developed and implemented in the COmmunity HEalth System InnovatiON (COHESION) Project, a global health project aimed at strengthening health systems in Mozambique, Nepal and Peru. This project developed innovative formative research at policy, health system and community levels to gain a comprehensive understanding of the barriers, enablers, needs and lessons for the management of chronic disease using non-communicable and neglected tropical diseases as tracer conditions. After formative research, COHESION adopted a co-creation approach in the planning of interventions. The approach included two interactions with each type of stakeholder at policy, health system and community level in each country which aimed to develop interventions to improve the delivery of care of the tracer conditions. Diverse tools and methods were used in order to prioritise interventions based on support, resources and impact. Additionally, a COHESION score that assessed feasibility, sustainability and scaling up was used to select three potential interventions. Next steps for the COHESION Project are to further detail and develop the interventions propositioned through this process. Besides providing some useful tools and methods, this work also highlights the challenges and lessons learned from such an approach.

**Summary box**

- There have been calls for improved methodological approaches to implementation research in global health.
- Co-creation approaches allow the development of interventions with local stakeholders playing an active role and are gaining traction in implementation research, but few practical approaches have been described in the global health literature.
- Prior to co-creation, formative research with a policy analysis, health systems assessment and community perception study using non-communicable diseases and neglected tropical diseases was completed to assess barriers, enablers and lessons for the management of these tracer conditions.
- The tools and methods used for co-creation need to be adapted according to the characteristics of the stakeholders and each setting.
- The principal challenges for researchers using this approach is the investment in time and resources in working closely with partners and not knowing which interventions may result.

## Introduction

The WHO highlights that ‘research is fundamental to generate knowledge and information for formulating evidence-informed policies and practices in support of global public health and health equity’^1^ showing the need for context-specific information to develop solutions to address the ‘know-do gap’.^2^ Knowledge translation promotes research uptake by different stakeholders through synthesis, dissemination, exchange and use of results to improve outcomes.^3^ Knowledge translation is influenced by the credibility of the research, which is impacted by the people involved, previous work in the field by the researchers, trusted local partners and the use of the results by knowledge brokers.^4^ Knowledge translation efforts tend to start from the point where research findings are ready for uptake and use with policy makers as their primary audience.^5–7^ This tends to neglect opportunities to ‘plant seeds’ for future uptake by placing emphasis at the design stage of research.

One element at the design stage that is often overlooked is the initial development of interventions, as these are often planned and implemented in an ad-hoc manner.^8^ Another critical element is the need to understand the context in which the interventions will be implemented. This includes the roles stakeholders play as well as factors ranging from political issues, use of health systems and local explanations of health problems.^9–11^

Although guidance on implementation research exists,^11–13^ there have been calls for improved methodological approaches in global health focusing on both conceptual factors and how interventions are implemented and evaluated specifically in low-income and middle-income countries.^14^ This paper details the process of developing research methods; sharing results with stakeholders and co-creating interventions with local stakeholders, thereby proposing an approach to develop interventions grounded in the local context and developed jointly by beneficiaries.

## Context and approach

The COmmunity HEalth System InnovatiON (COHESION)^15^ Project, funded by the Swiss Programme for Research on Global Issues for Development,^16^ aims through research, stakeholder engagement and targeted interventions to address, from a user-centred perspective, the response of primary healthcare (PHC) in meeting people’s health-related needs.

The COHESION project includes formative research at policy, health system and community levels using non-communicable diseases (NCD) and neglected tropical diseases (NTD) as tracer conditions for policy, health system and community responses^17–19^ in order to identify barriers to care for vulnerable populations. The tracer diseases were diabetes and hypertension (NCDs), and for NTDs, schistosomiasis in Mozambique, leprosy in Nepal and neurocysticercosis in Peru representing three very different health and socioeconomic contexts^20–22^ (see online supplementary appendix 1).

10.1136/bmjgh-2018-001183.supp1 Supplementary data

In each country two sites, a rural and an urban or periurban area were selected as being ‘representative’ by local stakeholders. The research protocols were submitted and approved by the following ethics committees:

- Commission Cantonale d'éthique de la recherche, Genève, Switzerland.
- Comité Institucional de Bioética em Saúde da Faculdade de Medicina/Hospital Central de Maputo, Maputo, Mozambique.
- Nepal Health Research Council, Kathmandu, Nepal.
- Institutional Review Board of the Universidad Peruana Cayetano Heredia, Lima, Peru.

### Policy analysis

At global and national levels, a range of activities, for example, the inclusion of NCDs and NTDs in the Sustainable Development Goals, have taken place in recent years and resulted in a stream of documentation and policy pathways. As part of the formative research, these were mapped and enabled an understanding of how NCDs and NTDs are addressed in both global and national political agendas. As discussed by Walt *et al*,^23^ this approach used a case study methodology, with a key document review at global and national level as well as key informant interviews. The analysis applied the framework developed by Shiffman and Smith.^24^

### Health systems assessment

COHESION’s formative research also included a health systems assessment building off expertise in developing^25 26^ and implementing health system assessments based on Rapid Assessment Protocols.^27–35^ A manual using this approach previously field-tested in Lima, Peru^36 37^ was adapted for NTDs. Mixed-methods data collection techniques were used, gathering secondary data (local literature, data from national statistics, regulations and so on) as well as primary data from observations and interviews. The assessment was carried out at 4 levels (national, regional, local and individual) and described 11 themes for the selected diseases (ie, Healthcare structure and organisations; Policy environment; Financing; Data collection and information systems; Healthcare workers; Service Delivery, etc.).

### Community perceptions study

Knowledge of community capacities is necessary to be able to improve healthcare in line with local needs and resources.^7^ Community mapping, in-depth interviews and focus groups were used to get a better understanding of the community’s perceptions on health problems, healthcare services and the impact of the tracer conditions at the individual, family and community level. Special attention was made to include the views of ‘marginalised’ groups, for example, members of different castes in Nepal and elderly people in Peru.

### Moving from research to interventions

The COHESION Project, used the WHO’s definition of intervention: ‘a health intervention is an act performed for, with or on behalf of a person or population whose purpose is to assess, improve, maintain, promote or modify health, functioning or health conditions.’^38^ COHESION adopted a co-creation approach, defined as the process of involving stakeholders in the development of the services and interventions^39^ in order to foster sharing and discussion around possible ways of addressing a specific issue. This aimed to have a better understanding of the context and how to address the specific problems identified,^39 40^ which some argue has a positive impact on the intervention and approach.^41^

COHESION’s approach to co-creation^42^ includes two interactions with each stakeholder group (policy, health system and community). Initial ideas for interventions were collected following the presentation of the formative research. The first meeting aimed to highlight the problems identified, justify these based on the findings and then propose a list of possible interventions. These interventions were then assessed by the COHESION team to identify the extent to which they were a priority for community, health system and/or policy stakeholders as well as their feasibility, sustainability and fit with the aim of the project.

**Figure 1:**
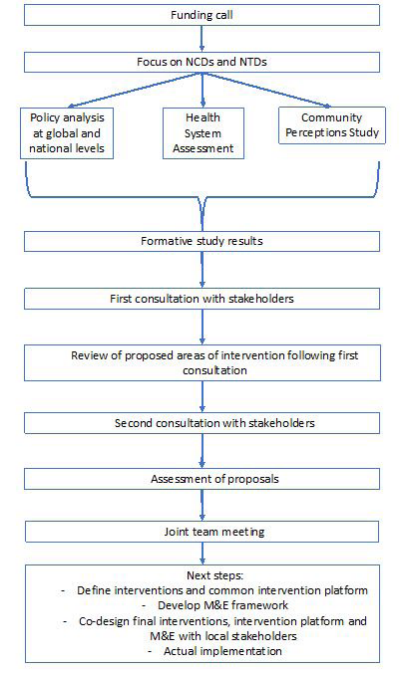
Overall steps in COHESION methods. NCD, non-communicable diseases; NTD, neglected tropical diseases; COHESION, COmmunity HEalth System InnovatiON.

Once the COHESION team ‘filtered’ the interventions, the remaining options were presented to stakeholders again and then they were asked to prioritise and evaluate their feasibility. For the health system and policy level stakeholders, a structured approach was taken using a tool for prioritisation of interventions during the second meeting (table 1). For the communities, a specific ‘prioritisation of interventions for communities’ tool was developed (table 2). The final step in the process involved each team in each country defining priorities for their context using a scoring tool (table 3). This overall process is presented in figure 1.

**Table 1:** Tool for prioritisation of interventions for health system and policy level stakeholders during second meeting

Institution or organisation the person represents:									
Problem to be addressed	List of interventions	Support			Resources			Impact	Total
		Part of mandate		Policy environment facilitates	Human	Financial	Expertise (please describe)		
		Institutional	Individual						
Problem 1	Intervention A	A1	A2	A3	A4	A5	A6	A7	=A1 x A2 × A3 × A4 × A5 × A6 × A7
	Intervention B								
	Intervention C								
Problem 2									

**Table 2:** Prioritisation of interventions for communities during second meeting (includes examples for discussion purposes)

Problem to be addressed	Intervention(s)	Ranking of this intervention by participants	Why is this important?	Why is this a priority versus other interventions?
Low quality of management of diabetes	Better access to diagnostic tools at PHC Healthcare worker training Training of nurses	2	Communities feel that diabetes is poorly managed	Priority due to high level of poor outcomes of people with diabetes
Lack of knowledge of NTDs	Media campaigns of NTDs Posters in community on selected NTD Use of community as educators on NTD	3	Communities feel that they do not have sufficient information on NTD to be able to address this health concern effectively	Priority as people are not able to address their own health concerns
Women feel overburdened with care of people with chronic diseases	Development of women’s groups Peer support networks Strengthening of PHC	1	Women are missing out on other opportunities due to burden of care	Priority as women feel that they are unable to address other roles and also be economically productive
Access to medicines for hypertension is poor	Training of pharmacists Coordination meetings with people responsible for supply systems	4	People diagnosed with hypertension are not able to access medicines and need to pay high prices for these in private sector	Priority as this impacts management of hypertension as well as being a financial burden on households

NCD, non-communicable diseases; NTD, neglected tropical diseases; PHC, primary healthcare.

**Table 3:** Tool for defining three interventions to be used by COHESION

Scoring criteria		Possible intervention 1	Possible intervention …
Disease and health system subscore	Does this intervention impact both NCDs and NTDs?		
	Can the intervention be implemented at PHC?		
	Does this intervention positively impact care delivery at PHC?		
	Does this intervention positively impact UHC?		
*Subscore*		*=(Total ÷ 4) × 40*	
Population subscore	Does the intervention impact adults only (0 points), children only (0.5 points) or all ages (1 point)?		
	Does the intervention positively address specific issues for vulnerable groups (poor, indigenous and so on)?		
	Does the intervention positively address specific gender issues?		
*Subscore*		*=(Total ÷ 4) × 40*	
Intervention subscore	Can the intervention be delivered for the budget available?		
	Is this intervention sustainable?		
	Is this intervention easily scalable?		
	Can the intervention be easily integrated into existing services?		
*Subscore*		*=(Total ÷ 4) × 20*	
TOTAL SCORE		= Sum of three subscores	

NCD, non-communicable diseases; NTD, neglected tropical diseases; COHESION, COmmunity HEalth System InnovatiON; PHC, primary healthcare; UHC, Universal Health Coverage.

## Application of this approach in three countries

This section describes the implementation of the co-creation process which took approximately 2 months and the adaptations made in each context.

### First consultation with stakeholders

In order to gain insight into possible interventions from policy level stakeholders, the Peru and Nepal teams used the opportunity provided by the interviews for the health system assessment, to ask additional questions. This was done to capitalise on the opportunity of interviewing people who usually have little ‘time to spare’ at the policy level in both contexts. The additional questions revolved around suggesting policies or projects that could be implemented to improve PHC for managing the target diseases.

In addition to these questions, a meeting in Kathmandu Nepal was organised with community medicine physicians and public healthcare workers to obtain the perspective of health system stakeholders. During the meeting, researchers presented the findings of the health system assessment and community perceptions study and then asked for suggestions of interventions. Additionally, input for interventions was collected during a meeting with the Nepal Health Research Council, where preliminary results were presented and participants were asked for suggestions for interventions. A similar meeting was organised in Peru for stakeholders at health system level.

In Peru, at the community level, a meeting was organised with the local population to provide information on selected diseases followed by group discussions to propose possible interventions. In addition, a meeting with healthcare workers at PHC level was held explaining the co-creation process and asking them to provide ideas for possible interventions. In Nepal, a joint meeting between the community and PHC workers was held. This meeting took a similar approach to that in Peru by providing information on selected diseases, including the findings of the health systems assessment with an emphasis on the problems from the community and followed by group discussion asking for any suggestions.

In Mozambique, the results were presented at meetings that were divided into policy, health system and community levels. PHC workers were included in the health system meeting. After the presentation, participants were asked for suggestions to improve the situation of people affected by NCDs or NTDs.

In the first consultation, different numbers of participants provided their suggestions in each country. Across actors, the number of suggestions ranged from 113 to 231 in Nepal, 10 to 84 in Peru and 6 to 11 in Mozambique. In both Nepal and Peru, the largest number of inputs came from the policy level stakeholders.

### Review of proposed areas of intervention following the first consultation

Between the first and second consultation, filters based on prioritisation by each stakeholder and feasibility were applied by the research teams in each country. In Peru, the process consisted of: systematisation of proposals according to implementation level, elimination of duplicates and a meeting with experts in the project’s region. In Mozambique, the team first eliminated duplicates and reviewed pros and cons of the interventions to select the most feasible ones. Nepal grouped similar interventions and removed duplicates from the list. This resulted in 6 interventions in Mozambique, 17 in Nepal and 7 in Peru.

### Second consultation with stakeholders

During the second consultation, a prioritisation process was conducted with the interventions selected after the first consultation. Policy and health system stakeholders were asked to use specific evaluation criteria (support, resources and impact) to assess interventions. Community level participants were asked to prioritise the interventions according to the importance for their community. In Peru, in order to prioritise interventions at the policy level, an email was sent to the participants of the first consultation. They were provided with the tool shown in table 1 along with information about the potential interventions and instructions on how to score each element for the intervention. At the health system level, the potential interventions were presented during a meeting. Afterwards, participants completed table 1. At the community level, a meeting was held with the community and PHC healthcare workers. First, potential interventions were described, afterwards in small groups, participants were asked to select the three most important interventions and the three least important interventions using the tool shown in table 2.

In Mozambique, only interventions that passed the internal filtering process were presented. In both Mozambique and Nepal, a more qualitative approach was taken with communities by explaining the potential interventions and asking them what they thought and felt about each of them. The Nepal Team presented all interventions listed by order of priority to policy level stakeholders. At the policy and health system levels, the potential interventions were presented and described and then the tool with evaluation criteria was used (table 1), similar to what was done in Peru. PHC workers were again integrated into the health system meeting. During the second round in Nepal, only PHC workers were included from the health system perspective. They were present at a joint meeting with the community where they ranked the filtered interventions according to importance and were asked to give a reason for their choice.

### Assessment of proposals

After the second consultation, every country had a different number of proposals. In Nepal, after the prioritisation, the top potential interventions were selected and (table 3) was used to calculate the COHESION score. In Peru, the interventions that were prioritised and those interventions with the higher COHESION Score were matched to select the interventions for each level. Mozambique applied the COHESION Score between the first and second consultation, so final areas of possible interventions at each level were defined after the prioritisation process.

All potential interventions were synthesised into three potential areas for intervention table 4) and discussed at a joint meeting across country teams and more information can be found on http://www.cohesionproject.info.

**Table 4:** Potential areas of intervention from each country

Level/Country	Mozambique	Nepal	Peru
Policy	Advocacy for increased focus on NCD and NTD at policy level	Engagement of policymakers for more focus on NCDs and a refocusing on leprosy	Management of health resources (financial, material) promoting coordination of national and regional level
Health System	Training for healthcare workers on case management of the three diseases including issues on gender and vulnerable populations	Improve the management of the three diseases at PHC level using synergies for prevention and management of complications.	Training of health personnel with guidelines to transfer knowledge to new workers
Community	Community health education on prevention and self-management of the three diseases	Awareness raising at community level for the three diseases promoting prevention and early detection	Training of community health workers with focus on health promotion and follow-up of patients

NCD, non-communicable diseases; NTD, neglected tropical diseases; PHC, primary healthcare.

### Next steps

Next steps are to further detail and develop the final interventions co-created through this process using the United Kingdom Medical Research Council guidelines.^3^ Anticipating the diversity of contexts and priorities, the interventions will be detailed using the TIDieR checklist.^43^ Once this is completed, the final stage will be to present this to stakeholders to fine-tune the interventions to the stakeholders’ specifications. For example, if patient education is going to be delivered, then who might be best suited to do this: nurses or trained peers, the frequency of this education and where it will be delivered also need to be determined.

## Lessons learned

The formative research approach is innovative in that it covers all levels of the health system from policy to the community enabling an overall view of the context in which interventions will be implemented. In addition, the use of tracers allows for an overall view of different elements of the health system. As the process of intervention development is integrated into the formative research this allowed the researchers to better understand the local context, for example, how a disease is understood and perceived by local communities. Using NCDs and NTDs as tracer conditions aims to ensure that responses developed do not increase competing demands on the health system, but rather enable NCD and NTD interventions to benefit the health system as a whole.^44^ Combining this research approach in three different countries and in both rural and urban areas allows for sharing experiences between contexts and for finding similarities and differences. Although approaches to implementation research are clearly described, for example, in the WHO Special Programme for Research and Training in Tropical Diseases, many do not provide guidance on carrying out a situational analysis.^11^

Involving local stakeholders early identified opportunities and challenges in the environments where the interventions will be implemented. In this process, stakeholders are first key informants, then active participants in intervention development and finally possible users of the interventions. Others have argued that this process allows for better ‘buy-in’ of the interventions and might impact their integration and sustainability in practice.^45^ This involvement leads to the issue of stakeholders’ availability and how to find different ways of contacting and engaging with them. An ethical challenge the COHESION project faced was that the funding mechanism provided funds in two phases and did not guarantee the second tranche of funding needed to implement the interventions until after a mid-term review. Thus, teams had to be very clear about this with all those involved in the formative research and co-creation activities that the actual implementation of interventions could or could not happen. In addition, the scope of COHESION limited the type of interventions that could be supported. This raises issues about co-creation processes and how to address interventions seen as priorities by stakeholders, but outside the confines of the given project. Another issue faced was how to involve and consider different views from policy makers, healthcare professionals and communities when developing interventions together. Although the COHESION Team developed specific tools to guide the ‘unbiased’ assessment of different interventions, a balance needed to be found between trying to evaluate possible interventions quantitatively with the tools provided and qualitatively based on the experience of the research teams and discussions on the needs and priorities of each stakeholder group.

It is important to reflect that knowledge is not the same for the stakeholders and their capacities to provide proposals are unequal. For these reasons, adaptation of messages was required by researchers when presenting the results to communities, individuals within the health system and those at policy level. This was facilitated in the COHESION project, as the team included health communication scholars and the funding allowed for substantial investments in communication processes. The funding mechanism also allowed for conducting formative research, intervention development and implementation without having these interventions predefined. This highlights the importance given for research to identify solutions and options for overcoming implementation obstacles taking into account the local context.^10^ The issue of priority setting is discussed in the literature; however, it focuses on how overarching issues get on the research agenda.^46–48^ For many researchers responding to calls for proposals, there is very little guidance on the way of being able to influence this.

Co-creation has become more widely used as it allows an alignment of research and intervention development to better address the needs of the targeted populations.^41^ A recent scoping review found that co-creation approaches are gaining traction in implementation research.^49^ However, this study did not include communities or possible users and highlighted the need to test this approach to developing interventions. The method described here will be of use to many researchers embarking on the development of interventions through a co-creation process by offering both a methodological approach to assess the context and then how to actively involve stakeholders in co-creating possible interventions with researchers.

## Conclusion

Besides providing some useful tools and methods, this work also highlights the challenges and lessons learnt from such an approach. Researchers need to think about the methodological aspects of the research and creation of interventions based on the best available evidence and also communication and engagement activities in order to foster partnership between stakeholders on the ground. In so doing, this project rejected a paternalistic view of defining research needs and priorities and rather generated and guaranteed spaces to gather, organise, prioritise and jointly define areas for intervention with all key stakeholder groups. This approach provides researchers with both challenges and opportunities. The challenges relate to investment in time and resources in working closely with partners and not knowing which interventions may result. On the flipside, this offers a unique opportunity to develop interventions together with local stakeholders and ensuring their buy-in from the start of the process.
