# Supplementary material for: Moving from formative research to co-creation of interventions: insights from a community health system project in Mozambique, Nepal and Peru
Source: BMJ Glob Health. 2018 Nov 16;3(6):e001183. doi: 10.1136/bmjgh-2018-001183 (PMC6254743; doi:10.1136/bmjgh-2018-001183)
Supplement: Supplementary data [file bmjgh-2018-001183supp001.pdf]

Appendix 1 – Background information on Mozambique, Nepal and Peru[47-49]

	Mozambique	Nepal	Peru
Total population (000)	25,834	27,797	30,376
Gross national income per capita (PPP)	1,040	2,260	11,360
Life expectancy at birth m/f (years)	52/54	67/69	75/79
Probability of dying (15-60 years; both sexes) per 1,000 population	466/453	197/164	118/91
Total expenditure on health per capita (Intl $)	66	80	555
Total expenditure on health as % of GDP	6.4	5.5	5.1
% of all deaths caused by NCDs	23%	60%	66%
Raised blood pressure (ages 20-74)	38.1%	24.2%	21.0%
Diabetes prevalence (ages 20-74)	2.5%	4.5%	4.3%
% of DALYs caused by NTDs	1.7	1.3	1.2
Human Development Index rank out of 187	178	145	82
